# Coworker Trust and Knowledge Sharing among Public Sector Employees in Kenya

**DOI:** 10.3390/ijerph17062009

**Published:** 2020-03-18

**Authors:** Felix Kipkosgei, Seung Yeon Son, Seung-Wan Kang

**Affiliations:** 1 College of Business, Gachon University, Seongnam 13120, Korea; felixkipkosgei@gmail.com; 2 Graduate School of Defense Management, Korea National Defense University, Nonsan 33021, Korea; faithnet153@naver.com

**Keywords:** coworker trust, team member exchange, knowledge sharing, task interdependence, technology

## Abstract

This study investigates the association between coworker trust and knowledge sharing among public sector employees with additional consideration of team-member exchange (TMX). It also accounts for the use of supportive technology as a determinant of coworker trust. The study aims to develop a framework to help organizations understand the complex associations among coworker trust, exchange, and knowledge sharing and recognizes the roles of supportive technology and task interdependence in those associations. A cross-sectional survey of 255 employees at three Kenyan public organizations was analyzed. A hierarchical regression analysis tested five hypotheses in eight models to estimate direct, moderating, and mediating relationships. Coworker trust was positively related to knowledge sharing and TMX. Supportive technology significantly moderated the relationships; however, task interdependence was not statistically significant. The results imply that organizations might increase knowledge sharing by focusing on building trustful bonds among workers.

## 1. Introduction

Knowledge is a vital organizational asset [1,2,3]. Thus, just like the management of other organizational resources, managing knowledge is important for an organization. Effective knowledge management has multiple benefits for an organization, including improved productivity, performance, and innovation capability [4,5,6]. Knowledge sharing is the most important knowledge management process [7]. In fact, Witherspoon et al. [8] identified knowledge sharing as a building block necessary for organizational success and is often adopted as a survival strategy. Research on knowledge sharing is highly relevant in the 21st century, and many specialized journals and conferences, including those that focus on management, have been established [9]. Although knowledge sharing in organizations is important to all the relevant stakeholders, intra-organizational knowledge is mostly not shared [10].

Knowledge sharing refers to the transfer of knowledge among individuals, teams, departments, and organizations [11]. Knowledge sharing is important to ensure a good fit between an organization’s mission and its vision, as well as for team accountability, team-work environments, and decentralized organizational decision-making [12]. Zhang et al. [13] pointed out that knowledge sharing enhances intra-organizational cooperation, which in turn leads to team effectiveness. Knowledge sharing is vital for employees as a platform for sharing their experiences [14]. Kim and Park [15] and Abbas et al. [16] linked knowledge sharing to innovation, which is highly desired by modern organizations.

In recent times, many organizations use technology to enhance their members’ knowledge-sharing abilities [14,17,18,19]. Although advanced information technologies (ITs) and networked systems are enhancing knowledge distribution, organizational members are the key actors in disseminating relevant knowledge [20]. Members are motivated to use these tools to share knowledge because doing so tends to advance their personal knowledge [14]. However, in contexts that do not fully capitalize on these tools, knowledge-sharing outcomes might be less significant, and, in cultural contexts that do not value knowledge sharing, organizational members might reject these technological systems [21]. 

Like most African countries, Kenya still lags behind, as far as knowledge management practices are concerned [22]. Mosoti and Masheka [23] noted that because knowledge management practices are not well understood by most public organizations in Kenya, the creation and implementation of knowledge management practices, including knowledge sharing, is often not a priority. Consistent with this argument, Ondari and Minishi [24] argued that management of state corporations in Kenya rarely offer training to their employees on new innovations and knowledge management processes and are often slow to adopt human resource management solutions, such as knowledge management [25]. Thus, this study is conducted within the context of the Kenyan public sector to gain a better understanding of the antecedents and the contextual factors that may impact knowledge sharing. 

In recent times, advanced technologies are gaining prominence in Kenya as enterprises and organizations aim to increase their creativity and innovation because of the persistently increasing competition of businesses in both the private and public sectors. Most of these organizations are trying to create new ideas that lead to innovation and the sustainability of their business models. Through Kenya Vision 2030 (launched in 2008, with a main goal to attain the status of a middle-income nation by 2030 [26]), Kenya is focusing on becoming a knowledge-driven state, and knowledge sharing is a crucial aspect of the Kenya Vision 2030 agenda for rapid economic growth. One manifestation of the agenda’s implementation is the development of startups, many of which need various types of support, including relevant ideas to enhance their creativity and innovation. Supporting them and their success in particular would be possible if the established and successfully growing enterprises prioritized sharing knowledge with each other.

Knowledge sharing may be stimulated by trust [27]. Trust is a multidimensional concept that encompasses three dimensions, including ability, benevolence, and integrity [28]. Competence trust denotes confidence (i.e., ability) regarding the exchange partner’s ability to behave or perform as expected, while goodwill trust focuses on the motives and intentions of the exchange partner to act in a fair and trustworthy manner (i.e., benevolence and integrity) [29,30]. Because the study hinges on the expectation that employees will show goodwill and share their knowledge if they perceive trust from coworkers, our study focuses on goodwill trust when we refer to trust.

This study aims to address several gaps in the literature. Foss [31] pointed out that a main management problem is knowledge sharing and advocated finding ways to encourage knowledge workers to develop sharing behaviors. To this end, previous scholars have advocated the use of the social exchange theory (SET) to improve the understanding of the conditions that influence knowledge sharing [32]. Using the SET [33], this study presents co-worker trust as an antecedent of knowledge and re-validates prior research that identified this link [27]. To provide further insight into knowledge sharing, Mayer and Gavin [34] asked for researchers to continue to examine the mechanisms through which trust affects knowledge sharing. Therefore, we present the quality of exchange in coworker relationships (also known as team-member exchange (TMX)) [35] as a mediating mechanism that may explain this relationship.

**Figure 1:**
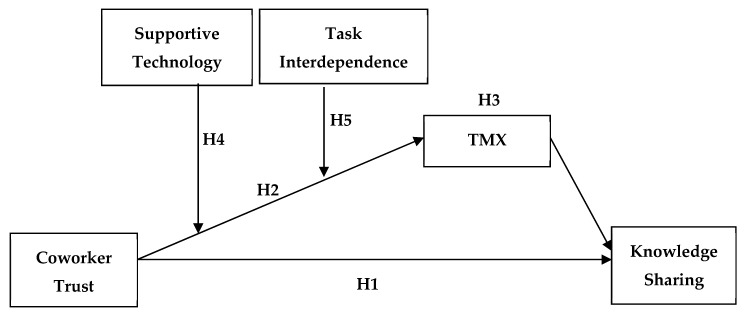
Hypothetical model. TMX: team-member exchange.

In a recent meta-analytic review, De Jong, Dirks, and Gillespie [36] revealed the need for further research into potential moderators of trust to ensure a more comprehensive framework of trust. With the advancement in technology, many companies are beginning to incorporate technology into their organizational processes. Thus, we focus on supportive technology as potential boundary conditions of trust. In addition, we investigate the possibility of task interdependence, a critical element of team performance, as a boundary condition of trust. It is worth noting that the majority of knowledge sharing studies from non-Western cultures have been conducted in Chinese cultures [32]. Wang and Noe [32] call for more research in emerging economies in countries in Africa, the Middle East, and South America to get a better understanding of how knowledge sharing may differ due to cultural differences. The study attempts to respond to this call by using a cross-sectional survey comprising of Kenyan public sector employees. The hypothesized research model is presented with Figure 1.

In the following sections, we review the literature linking trust to knowledge sharing and TMX, explore the boundary conditions of trust, and present the study hypotheses. We then explain the data collection method and procedure and present the results of our analyses (e.g., confirmatory factor analysis (CFA), correlation, regression, and the bootstrapped indirect effect test). Finally, we discuss the theoretical contributions and practical implications and end with the limitations and agenda for future research. 

## 2. Theoretical Background and Hypotheses

### 2.1. Coworker Trust and Knowledge Sharing

Mayer, Davis, and Schoorman [28] defined trust as the “readiness to accept the influence of another party’s actions based on the belief that the other party will accomplish a particular task relevant to the trustor, irrespective of the ability to monitor the other party” (p. 712). Trust has been conceptualized as a vital organizing principle through which organizations allocate and coordinate their activities [37]. In particular, McEvily, Peronne, and Zaheer [37] suggest that trust influences organizations by informing how employees interact and motivates employees to contribute and combine knowledge resources. Due to the important role trust plays in an organization, trust has been linked to multiple positive workplace outcomes at the individual, team, and organizational level [38,39,40,41].

SET emphasizes that before individuals engage in transactions, they make a calculated assessment of costs and benefits of their actions before engaging in an act [33]. Thus, people are motivated to act in a particular way if they believe that the potential benefits can outweigh the potential costs. SET proposes six types of resources that individuals may exchange. They include love, money, status, goods, services, and information (or their knowledge) [42]. A core concept of goodwill trust is that other people will be fair and act with good faith in their interaction [29,30]. This supports the argument that when coworkers establish trust with each other, they would be more willing to share knowledge because there is an expectation that, when required, the receiving party will reciprocate. 

There have been multiple studies that support the impact that trust has on knowledge sharing. For instance, Holste and Fields [27] found that affect-based trust (grounded in mutual concern for others) and cognition-based trust (grounded in coworker competence and reliability) factor in staff member decisions to share knowledge that cannot be formally documented. Bakker et al. [43] advanced the literature by examining the three dimensions of trust and found that employees share more information with coworkers they perceive honest and fair and share less information with coworkers they perceive as very capable. The research found no significance between the benevolence dimension of trust and knowledge sharing. Using a sample of virtual teams, Pinjani and Palvia [44] argued that trust is an effective way for employees to open up and share information, especially when employees are unable to meet face-to-face.

With regards to knowledge management processes in general, Shapin [45] found that trust had a vital role in knowledge distribution. In addition, Chai and Kim [46] found that workplace trust encouraged effective knowledge transfers and exchanges, and Nonaka [47] found that interpersonal trust was essential for developing a knowledge-sharing organizational environment. Mayer, Davis, and Schoorman [28] proposed that workers readily listen to each other and are more likely to absorb the knowledge being shared when trust exists. That is, individuals are more likely to recognize the value and apply the new knowledge when the giver is someone they trust [48]. Following extant literature that support this rationale [49,50], the following hypothesis was developed.

**Hypothesis** **1 (H1):** *Trust among coworkers positively influences knowledge sharing.*

### 2.2. CoworkerTrust and Team-Member Exchange 

Farmer, Van Dyne, and Kamdar [51] pointed out that workers who offer support to others tend to receive information, recognition, and assistance from reciprocating workers. Al Hosani, Elanain, and Ajmal [52] defined TMX as reciprocal relationships among team members in any work environment in which team members mutually share support, feedback, and ideas relevant to optimal performance. Moser [53] found that trust within a team enhances TMX. A strong sense of belonging, group identity, and cooperation ensues among employees in high TMX relationships due to the strong cohesion within the team [54]. Using a SET framework, Schermuly and Meyer [54] indicated that interpersonal relationships and social interaction at the workplace constitute better interpersonal communication. Chiu, Hsu, and Wang [55] indicated that workplace friendships that develop from trust lead to high quality TMX, commitment, reciprocity, common interests, and shared values.

Haynie [56] interpreted social exchange as typified by long-term non-specific obligations, in response to which individuals willingly interact with others at personal and professional levels. Those obligations encourage workers to establish better relationships, and reciprocal ties tend to establish strong interpersonal bonds. The norm of reciprocity indicates that improved work relationships, unlike negotiations, allow for employees to develop trusting and committed relationships with one another [57]. SET contend that because such interactions tend to be interdependent (or contingent on the actions of others) [33], these interactions may generate high-quality relationships [42]. Therefore, it is logical to assert that coworker trust positively relates to the quality of relationship with coworkers.

**Hypothesis** **2 (H2):** *Trust among coworkers positively relates to Team-Member Exchange.*

### 2.3. The Mediating Role of Team-Member Exchange

Interpersonal relationships among coworkers are crucial to how employees behave at work. Andrews and Delahaye [58] argued that the effectiveness of a team’s relationships might be understood by examining TMX, which they defined as the quality of the team members’ relationships, where quality refers to reciprocity of ideas, resources, feedback, and recognition. They proposed that when TMX is high, all member contributions are known to all other members. Employees in high TMX relationships are willing to share knowledge, provide feedback, and help other members. Liu et al. [59] proposed that TMX increases employee commitment to the team. Liden, Wayne, and Sparrowe [60] pointed out that team members with high TMX have more opportunities to share knowledge and knowledge resources, whereas team members with low TMX experience fewer ideological exchanges needed to accomplish tasks. Thus, high TMX promotes exchanges of skill and knowledge in teams and within the organization in general.

Knowledge sharing is the activity of an organizational member with unique valuable information to share with other members to benefit them and the entire organization [61]. This knowledge sharing is a communicative process between two or more parties who intend to exchange experiences and expertise [62]. Nerstad et al. [49] argued that the willingness to share knowledge is strongly influenced by trust. However, willingness is somewhat mitigated by knowledge bearers who believe that they own their knowledge and have the right to determine the extent to which they will share it. According to the SET, relationships evolve over time into loyal and mutual commitments [33]. We contend that high TMX relationships create environments of mutual reciprocity that offer opportunities to share knowledge. Therefore, knowledge bearers might be more willing to share knowledge with individuals who they share high TMX with. Thus, given that previous studies has shown a link between trust and building relationships [63], and TMX has been linked to knowledge sharing [59], we posit that TMX may play a mediating role. Therefore, the following hypothesis was derived.

**Hypothesis** **3 (H3):** *Team-Member Exchange mediates the relationship between coworker trust and their knowledge sharing.*

### 2.4. The Moderating the Role of Supportive Technology

Extant literature indicates that information technology, such as electronic forums, intranet, websites, and bulletin boards, facilitate effective knowledge sharing in and outside the organization [64]. One important function of a technology should be its ability to promote socialization within a team [65]. For instance, Sarker, Valacich, and Sarker [66], in developing a model of technology adoption by groups, noted that to determine the group supportability of a technology, the indicators to look for should include how the technology can increase transparency, sociality, and parallelism within the group. We use supportive technology to describe modern technologies that a work team perceives to support their group processes and outcomes, including team task performance [66]. Thus, a technology can defined as supportive in so far as the technology can support transparency and interrelationships among coworkers. 

Kramer [67] argued that a technology may promote trust only to the extent that it optimizes compliance with organizational rules. Similarly, Branscomb and Thomas [68] have advocated for software engineers to develop user-friendly software products aiming to ensure acceptance, supportability, and easy use; factors that are characteristic of supportive technology. King [69] emphasized that modern technologies that address user needs are vital to the success of these systems. Therefore, this study tested the possibility that the interaction between trust and employee perception that the work team’s technology is supportive may positively impact TMX and posited the following hypothesis.

**Hypothesis** **4 (H4):** *Supportive technology moderates the relationship between coworker trust and Team-Member Exchange, such that the relationship is stronger when there is high supportive technology.*

### 2.5. The Moderating Role of Task Interdependence

Task interdependence is the extent to which workers mutually depend on one another in order to accomplish a task [44]. Task interdependence is a way to ensure that individual team members view their individual contribution as vital to the team’s success, doing so is important, as perceived instrumentality of one’s own contribution is a reason for motivational loss in teams [70]. Existing literature suggest that task interdependence has an effect on trust [71] and developing trust requires opportunities to interact and exchange information [71]. Wilson et al. [72] found that the need for communication creates an environment that facilitates the development of trust. When there is high task interdependence, communication and mutual reliance is high, which can improve TMX. 

The rules of reciprocity of the SET suggest that interdependence promotes reciprocity. This is because interdependence results in bi-directional transactions, where something is given and received [42]. Similarly, Molm [73] points to interdependence as a defining characteristic of social exchange because interdependence facilitates cooperation and lowers risk. We contend that in a context of high task interdependence, the high dependence will result in improved TMX, especially when there is trust. However, when task interdependence is low, dependence on others is relatively weak and expectations for reciprocity may be low.

Dirks and Ferrin [74] suggested that the role of trust varies depending on the situational structure (i.e., the amount of uncertainty or ambiguity in an event). Task interdependence facilitates better relations among team members because when there is more interaction when tasks are interlinked, employees need to learn from each other to accomplish the tasks. Kozlowskiet al. [75] found that task interdependence impacts team processes, since it shapes the coordination requirements and the roles that team members play. On the basis of previous studies, we posit that task interdependence will moderate the relationship between trust and TMX.

**Hypothesis** **5 (H5):** *Task interdependence moderates the relationship between coworker trust and Team-Member Exchange, such that the relationship is stronger when task interdependence is high.*

## 3. Methods

### 3.1. Study Design and Sampling Procedure

To test the study hypotheses, a cross-sectional survey was conducted from white-collar workers from three Kenyan public sector organizations, who were recruited through convenience sampling. We selected organizations with employees who often share and use knowledge from colleagues in the discharge of their duties. The organizations operated in diverse industries: a tertiary institution, hospital, and national bureau of statistics. First, research assistants contacted the senior managers of the three organizations. The study was explained, and consent was obtained for employees to participate in the study. The sample included information technology (IT) professionals in the IT department of the tertiary institution (41%), nurses in the hospital (28%), and employees of a national bureau of statistics (31%). The sample consisted of employees working in areas such as IT, health care, and research. These employees often share and use knowledge from others in the discharge of their duties. With the assistance of human resource personnel in each organization, the research assistants administered the questionnaire to the employees. The questionnaire included a cover letter explaining the study’s purpose and a written consent form. The cover letter emphasized that participation was anonymous, confidential, and the data would be used only for research purposes. Altogether, 300 questionnaires were distributed at the three organizations, and 255 valid questionnaires were returned (85% response rate).

The respondents’ mean age was 35 years and 61% of the sample was male. About 5% of the sample had a high school education, 36% had a college degree, 47% had a university degree, 10% had a master’s degree, and 2% had a doctoral degree. The mean tenure at the current organization was 7.9 years.

### 3.2. Measures

Response options of the items used in all the variables were on a five-point Likert-type scale, where 1 = *strongly disagree*, through 5 = *strongly agree*. The complete items are presented in Appendix A.

#### 3.2.1. Coworker Trust

Coworker trust was measured with the responses to the four-item coworker trust scale (α = 0.70) adapted by Pinjani and Palvia [44]. A sample item is “I can depend on other members of my team.”

#### 3.2.2. Team-Member Exchange 

TMX was measured using the three-item scale (α = 0.70) devised by Chae, Seo, and Lee [76]. A sample item was “Other members of this team recognize my potential.”

#### 3.2.3. Knowledge Sharing

Knowledge sharing was measured using a three-item knowledge-sharing scale (α = 0.74) developed by Connelly and Kelloway [77]. A sample item is “We share one’s expert knowledge.”

#### 3.2.4. Supportive Technology

Supportive technology was measured using a six-item scale (α = 0.60) developed by Sarker, Valacich, and Sarker [66]. A sample item was “Technology enables the development of social relationships among team members.”

#### 3.2.5. Task Interdependence

Task interdependence was measured using a three-item scale (α = 0.72) developed by Campion, Medsker, and Higgs [78]. A sample item was “Within my team, the job performed by team members is related to one another.”

#### 3.2.6. Control Variables

Prior studies suggest that demographic variables may impact knowledge sharing behavior [79]. Specifically, Lazazzara and Za [80] found that older employees in the public sector experience lower explicit knowledge sharing, while Sarti [81] asserts that organizational tenure influences the knowledge sharing attitude of employees. Thus, following previous knowledge sharing literature [27], we controlled age, gender, and tenure.

#### 3.2.7. Common Method Bias

To assess the effects of common method bias (CMB) in the study, we followed MacKenzie, Lee, and Podsakoff [82] and performed Harman’s single-factor test. All the items used in the analysis were simultaneously subjected to an exploratory factor analysis. The result indicated that no single factor explained more than 19% of the covariance among the variables. Based on this analysis, we determined that the possible CMB is not serious in the study.

### 3.3. Analytic Strategy

Before performing the analysis, all the variables used in the interaction terms were mean-centered, and variance inflation factor (VIF) values were calculated. The results revealed that all the VIF values were less than 10, indicating that multicollinearity did not present a problem for the analyses [83]. Then, a confirmatory factor analysis was performed to assess the distinctiveness of the study variables, and the Chi-square difference test was performed using STATA 14.1. (Data Analysis and Statistical Software, Stata Corp., College Station, TX, USA). The model (Figure 1) of the hypothesized direct and indirect effects (Hypotheses 1 through 5) was tested using hierarchical multiple regression analysis. For the indirect effects, bootstrapping was employed to test the mediating effects [84]. 

## 4. Results

### 4.1. Confirmatory factor Analysis and Chi-Square Difference Test

The comparative fit index (CFI), the Tucker Lewis index (TLI) (cutoff values ≥0.95), and the root mean square error of approximation (RMSEA) (cutoff value ≤0.05) were used to assess the model fit [85]. The results indicated that the hypothesized model had the best fit (χ^2^ = 173.78, df = 136; CFI = 0.95; TLI = 0.93; and RMSEA = 0.03). For further verification of the hypothesized measurement model, we set alternative models and conducted the chi-square model difference test between the hypothesized model and the alternative models. The results (Table 1) show that all the alternative models were significantly different from the hypothesized models and the hypothesized model showed the best goodness of fit indicators.

### 4.2. Descriptive Statistics

Table 2 presents the means, standard deviations, and correlations of the variables in the study.

### 4.3. Hypothesis Testing

Hypothesis 1 (Trust among coworkers positively influences knowledge sharing) and Hypothesis 2 (Trust among coworkers positively relates to TMX) predicted positive relationships. Table 3 shows the results of the hierarchical regression analysis, in which TMX and knowledge sharing were the dependent variables. Coworker trust had a significant positive influence on knowledge sharing (b = 0.40, *p* < 0.001; Model 7) controlling for the effects of age, gender, and tenure, which supported Hypothesis 1. There was also a significant positive influence of coworker trust on TMX (b = 0.21, *p* < 0.01; Model 2), which supported Hypothesis 2.

To test Hypothesis 3 (TMX mediates the relationship between coworker trust and their knowledge sharing), a bootstrapping analysis using 5000 samples was performed to determine whether coworker trust indirectly influenced knowledge sharing through TMX [84]. The confidence interval (CI) of the indirect effect of coworker point estimates was 0.05, and 95% CI (0.012, 0.096) excluded 0. The results indicated a significant indirect effect of coworker trust on knowledge sharing through TMX. The unstandardized coefficient was 0.05 and the bias-corrected bootstrapped 95% CI did not contain 0(0.016, 0.104), which supported Hypothesis 3. 

**Figure 2:**
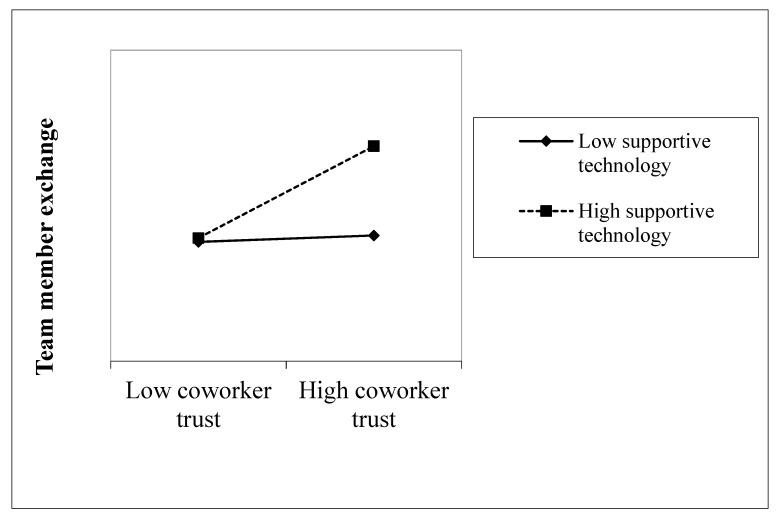
Moderating effect of supportive technology on the relationship between coworker trust and TMX.

Hypothesis 4 (Supportive technology moderates the relationship between coworker trust and TMX, such that the relationship is stronger when there is high supportive technology) concerned the moderating influence of supportive technology. To test Hypothesis 4, an interaction term was computed between supportive technology and coworker trust. The results in Table 3 (Model 3) show that the interaction term was positive and statistically significant (b = 0.17, *p* < 0.05). Figure 2 illustrates the results of a simple slope analysis that shows the relationship between coworker trust, TMX under the high supportive technology condition was statistically significant (b = 0.37, SE = 0.08, *p* < 0.001), and that the relationship was not statistically significant when supportive technology was low (b = 0.10, SE = 0.07, *p* = 0.16). Because the simple slope under the high supportive technology condition was significant, and the two simple slopes were different, the results provided further support for Hypothesis 4.

The result of the test of Hypothesis 5 (Task interdependence moderates the relationship between coworker trust and TMX, such that the relationship is stronger when task interdependence is high) is shown in Table 2. An interaction term was computed between task interdependence and coworker trust. The result in Model 4 was statistically non-significant (b = 0.06, *p* = 0.38), and Hypothesis 5 was not supported.

## 5. Discussion

There is increasing attention being paid to the role of trust in improving knowledge sharing in the workplace. In response to a call to pay more attention to knowledge sharing in emerging economies [32], this study conducted the study in Kenya. Specifically, we investigated and found support for TMX as the psychological mediating mechanism that explains the link between trust and knowledge sharing. Furthermore, we examined the moderating influences of supportive technology and task interdependence on the relationship between coworker trust and TMX, but there was no significance for the moderating role of task interdependence.

### 5.1. Theoretical Contributions

This study makes several theoretical implications to the literature. This study contributes to the knowledge management and knowledge sharing literature because it revealed antecedents of knowledge sharing. In a review, Wang and Noe [32] concluded that trust is an antecedent of knowledge sharing that needs further research attention. This study re-validates previous literature that found a positive significant relationship role of trust in influencing knowledge sharing [49,50] in an emerging economy (i.e., Kenya). The results are significant as it shows that even in a culture where management does not make knowledge management processes a priority [23,24], the link between trust and knowledge sharing still holds. In addition, a majority of the literature tends to focus on the direct relationship between trust and knowledge sharing without much attention to the mediating factors that explain this relationship. 

Using the reciprocity rule of the SET [33], this research presented TMX as the mediating mechanism that explains this relation. The results suggest that coworker trust and knowledge sharing are linked because of the nature of trust as a reciprocal interactive process, as elucidated by the SET. These findings empirically support the previous studies’ claims that trust is essential to relationship building [53] and knowledge exchange at work [32]. The modern workplace is becoming global and defined by a diverse workforce and virtual teams. Effective technology is a tool that could strengthen the positive effects of diversity and mitigate the negative impact of cultural diversity [44]. Prior research has established that technology is important for virtual teams to improve communication and quality of relationships among team members, team commitment, and team effectiveness [86]. Technology facilitates inter-personal processes like socialization and reduces conflict [44,65].

As expected, supportive technology played a moderating role in the relationship between trust and TMX. Our results align with prior literature from Pinjani and Palvia [44], who contend that when there is more electronic interaction between team members, this gradually influences the team’s attitudes and feelings. However, the results did not find significant results for the moderating role of task interdependence. The non-significance may be due to the diversity of the sample. For example, the nurses in the sample tend to work on rotation and tend to work with a different set of nurses in a new shift or sometimes even in a different department. Because of the mediating role that TMX plays between coworker trust and knowledge sharing, it is important that attention is paid to contextual variables that influence TMX. The significance of high supportive technology for the relationship between coworker trust and TMX suggest that by creating an environment that promotes supportive technology, this may inadvertently facilitate knowledge-sharing behavior as the technology improves the interaction and sociability within the team.

### 5.2. Practical Implications

The results of this study have practical implications for organizations and their managers. Organizations that heavily rely on teams should invest in technologies that support team processes, because coworker trust may be promoted through workers’ use of the technology. The increased trust would likely improve the quality of relationships between team members. The improved interaction would in turn increase knowledge sharing, which would benefit the organizations. Most organizations currently use modern technologies. Thus, the results of the study suggest that decision-makers, such as managers, need to ensure that the technologies employed focus on support capabilities, such as transparency, sociality, and parallelism that improve interrelationship among employees, doing so can promote their use as well as enhance team processes. Managers should promote supportive technology by implementing training to give their workers mastery and guidance. Developers should also concentrate on user-friendly technologies that make work easier for team processes.

To improve task interdependence, managers may need to design tasks in a way that requires workers to coordinate and work closely together, within which team members feel that their personal accomplishments have strong implications for the success of other team members [87], doing so may increase the social pressure to work harder, especially when efforts are low. Indeed, experimental research has found that people put in extra efforts when they believe their individual poor performance may inhibit other team workers [88].

The implication of the mediating role of TMX points to the important role that coworker relationships play in knowledge sharing. Therefore, managers need to focus on building coworker relationships and nurture their team-based environment by organizing events and activities that promote coworker interactions to build trust and respect. These activities should extend beyond orientation sessions at the early stage of team development and continue as part of the teams’ regular routine. Finally, due to important role that TMX plays in knowledge sharing, managers may want to recruit or assign employees with personality traits, such as openness to experience, extraversion, and agreeableness to positions or roles that require consistent knowledge sharing.

### 5.3. Limitations and Future Research

One of this study’s limitations is its potential for common method bias caused by the self-rated questionnaire. The results should be interpreted with caution because the respondents might have been biased for various reasons, even though Harman’s one-factor test suggests that common method bias may not be an issue in the results. The methodology for the data collection was convenience sampling using a cross-sectional design, which means that causal conclusions cannot be drawn from the results. Ina addition, the study was conducted at an individual level because it focused on worker perceptions and opinions. However, knowledge sharing was originally theorized as a group-level construct. Thus, future research should consider team-level or multi-level studies. Although the relationship between coworker trust and knowledge sharing might depend on cultural background, we did not directly examine the role of culture in impacting the hypothesized relationships. Finally, the present study used data from the Kenyan public sector. Thus, the results of the study may only be applicable to the public sector limiting its generalizability. Because the presence of multinational corporations is increasing in Africa, it is important to replicate this study on samples outside Kenya to get a better understanding of antecedents and factors that promote knowledge sharing in other contexts.

## Figures and Tables

**Table 1:** Model fit statistics for measurement models (*n* = 255).

Measurement Model	X^2^	Df	ΔX^2^	Δdf	TLI	CFI	RMSEA
Hypothesized	173.78	136			0.93	0.95	0.03
Three factors ^a^	196.80	143	23.02 **	7	0.91	0.92	0.04
Two factors ^b^	211.84	145	38.06 ***	9	0.89	0.90	0.04
One factor ^c^	214.83	146	41.05 ***	10	0.88	0.90	0.04

** *p* < 0.01, *** *p* < 0.001. ^a^ Knowledge sharing, coworker trust, and supportive technology were merged. ^b^ Knowledge sharing, coworker trust, supportive technology, and team-member exchange (TMX) were merged. ^c^ All variables were merged. TLI = Tucker–Lewis Index; CFI = comparative fit index; RMSEA = root mean square error of approximation.

**Table 2:** Descriptive statistics and correlations (*n* = 255) ^a^.

Variable	Mean	SD	1	2	3	4	5	6	7
1	35.35	8.78							
2	1.39	0.49	0.02						
3	95.14	76.44	0.80 ***	0.01					
4	3.46	0.74	0.06	−0.01	−0.01				
5	3.71	0.83	−0.02	0.00	−0.01	0.34 ***			
6	3.59	0.76	−0.05	0.03	0.06	0.29 ***	0.31 ***		
7	3.61	0.64	−0.06	−0.02	−0.05	0.39 ***	0.46 ***	0.30 ***	
8	3.66	0.77	−0.18 ***	−0.02	−0.12 **	0.49 ***	0.52 ***	0.34 **	0.52 ***

** *p* < 0.01, *** *p* < 0.001. ^a^ 1 = Age, 2 = Gender (1 = male, 2 = female), 3 = Tenure (in months), 4 = Task interdependence, 5 = Knowledge sharing, 6 = Team member exchange (TMX), 7 = Supportive technology, and 8 = Coworker trust.

**Table 3:** Results of hierarchical multiple regression analysis of the effects of the study variables on team member exchange and knowledge sharing; unstandardized coefficients (*n* = 255).

Variables	Team Member Exchange				Knowledge Sharing			
	Model 1	Model 2	Model 3	Model 4	Model 5	Model 6	Model 7	Model 8
Intercept	3.57 ***	1.61 ***	2.91 ***	2.89 ***	2.91 ***	3.82 ***	0.63 **	0.86 **
**Control variables**								
Age	0.00	0.00	0.00	0.00	0.00	−0.01	0.00	0.00
Gender	0.05	0.06	0.05	0.06	0.05	0.00	0.01	0.01
Tenure	0.00	0.00	0.00	0.00	0.00	0.00	0.00	0.00
**Independent variables**								
Coworker trust		0.21 **	0.22 ***	0.21 **	0.22 **		0.40 ***	0.48 ***
Task interdependence		0.15 *	0.16 *	0.15 *	0.16 *	0.34 ***	0.16 **	0.15 **
Supportive technology		0.15 *	0.18 **	0.20 *	0.18 *			
**Interactions**								
Supportive technology × coworker trust			0.17 *		0.18 *			
Task interdependence × coworker trust				0.06	−0.01			
**Mediator**								
TMX								0.14 *
F	0.44 **	7.71 ***	7.40 ***	6.72 ***	6.45 ***	0.06 ***	20.50 ***	18.48 ***
R^2^	0.01	0.16	0.17	0.18	0.17	0.11	0.32	0.33
R^2^ change		0.15	0.01	0.01	0.03		0.21	0.01
F change		14.92 ***	4.82 *	0.77	0.92		40.91 ***	36.89 *

* *p* < 0.05, ** *p* < 0.01, *** *p* < 0.001, two-tailed tests of significance.

## Appendix A

1. *Coworker trust [44].*
  1. Overall, the people in my team are very trustworthy.
  2. We are usually considerate of one another’s feelings on this team.
  3. The people in my team are friendly.
  4. I can rely on other members of my team.
2. *Team-Member Exchange [76].*
  1. I often ask others for help.
  2. Members of this team willingly suggest better work methods to others.
  3. Other members of this team recognize my potential
3. *Supportive Technology [66].*
  1. Team members are equipped with adequate tools and technologies to perform their tasks.
  2. Technology enables team members to work on different subtasks simultaneously.
  3. Technology enables team members to view other’s work whenever mutually desirable.
  4. Technology enables team members to modify other members’ work whenever desirable.
  5. Technology enables the development of social relationships among team members.
  6. Technology enables the sharing of knowledge among team members.
4. *Task interdependence [78].*
  1. I cannot accomplish my task without information or materials from other members of my team.
  2. Other members of my team depend on me for information materials needed to perform their task.
  3. Within my team, the job performed by team members is related to one another.
5. *Knowledge Sharing [77].*
  1. We share one’s ideas openly with each other.
  2. We share critical information about the project with each other.
  3. We share one’s expert knowledge.
